# Microfluidic ratio metering devices fabricated in PMMA by CO_2_ laser

**DOI:** 10.1007/s00542-020-04902-w

**Published:** 2020-06-01

**Authors:** M. Tweedie, P. D. Maguire

**Affiliations:** NIBEC, Ulster University, Belfast, BT37 0QB Northern Ireland, UK

## Abstract

We describe microfluidic fabrication results achieved using a 10.6 μm CO_2_ engraving laser on cast PMMA, in both raster and vector mode, with a 1.5″ lens and a High Power Density Focussing Optics lens. Raster written channels show a flatter base and are more U-shaped, while vector written channels are V shaped. Cross-sectional images, and, where possible, stylus profilometry results are presented. The sides of V-grooves become increasing steep with laser power, but broader shallower channels may be produced in vector mode by laser defocus, as illustrated. Smoothing of raster engraved channels by heated IPA etch, and transparency enhancement by CHCl_3_ vapour treatment are briefly discussed. An asymmetric Y meter is discussed as one method of diluting acid into seawater for dissolved CO_2_ analysis. Alternatively, microfluidic snake channel restrictors of different lengths in 2 channels may achieve the same result. Samples are fabricated with bases bonded by CHCl_3_ vapour treatment, and the devices are flow tested with either dilute food dye or DI water. Microfluidics fabricated in this manner have applications in ocean sensing of dissolved CO_2_ and other analytes, as well as broader sensing measurements, including biomedical sensors.

## Introduction

Many sensing applications require miniaturisation of sample fluid volumes in microfluidics systems, this being of interest for both environmental monitoring and biomedical purposes. Possible platforms include tethered and submersible probes, and onboard-ship analysis systems, for oceanic sampling of various analytes. These include phosphorus and phosphates (Bowden and Diamond 2003; Legiret et al. 2013), nitrites and nitrates (Beaton et al. 2012; Czugala et al. 2013), ammonium ion (Plant et al. 2009), manganese (Provin et al. 2013), ocean acidification via pH (De Vargas Sansalvador et al. 2016), and total dissolved inorganic carbon (Millero 2007).

Methods of measuring dissolved CO_2_ include conductimetric (Hall and Aller 1992; Sayles and Eck 2009; Bresnahan and Martz 2018), mass spectrometry (Bell et al. 2011), infrared gas analyser (Bass et al. 2012; Fassbender et al. 2015), and spectrophotometry (Nakano et al. 2006; Wang et al. 2013; Liu et al. 2013; Wang et al. 2015). A submersible platform which may, ultimately, incorporate dissolved CO_2_ sensing, is the Argo probe system, where a network of several thousand of these is currently deployed in the oceans for salinity, pressure and temperature depth profiling (Roemmich et al. 2009).

PMMA is a preferred material for many microfluidic systems because of its machinability, low cost, resistance to a wide range of chemicals, optical transparency, and the ability to thermally bond to itself to create sealed channels. It can be also bonded via an aminosilane interlayer to gas permeable PDMS, for gas transport from a liquid sample to another phase, e.g. gas carrier, vacuum or analyte solution, for determination of dissolved analyte gas in the sample (Tweedie et al. 2019). For dissolved CO_2_ measurement, for example, an acid is mixed with a water sample to liberate CO_2_, which then passes through a gas permeable membrane into an alkaline solution. There, the CO_2_ reacts with the OH^−^ ion, forming HCO_3_^−^, which has a lower ionic mobility than OH^−^, so reducing the solution conductivity, and enabling the CO_2_ content to be measured electrically. We have recently tested this for 2 mM CO_2_ detection, generated by acidification of 2 mM NaHCO_3_ solution, and detected by complimentary impedimetric and conductimetric measurements (Tweedie et al. 2020a).

Various methods of microfluidics production in thermoplastics such as PMMA have been demonstrated, including micromilling (Guckenberger et al. 2015), hot embossing (Mizuno et al. 2004; Jensen et al. 2004), and laser micromachining (Cheng et al. 2004; Hong et al. 2010).

Hot embossing of a master stamp under applied heat and pressure, preferably in vacuum to exclude air pockets, is useful for producing multiple replicates, but a typical embossing run can take from 30 to 60 min, depending on acceptable heating and cooling cycles. To change the microfluidic pattern, a new stamp must be manufactured, this being a time-consuming step, itself requiring, for example, micromilling of a master in brass (Ziyara et al. 2015), or photolithographically defining a stamp directly in Si (Mizuno et al. 2004) or in SU8 photoresist on Si (Mathur et al. 2009). Vacuum hot embossers are expensive, typically costing ~ £100 k, for floor-standing models.

Desktop micromills can produce high quality channels with vertical sidewalls, and this method works well for low density channels of 1 mm width, and up to 500 µm depth. However, with uncooled cutting bits in desktop micromills, the high thermal and mechanical stresses lead to frequent tool failures for bit diameters less than approximately 250–300 µm. Therefore, for complex microfluidic patterns with narrow or deep channels, the movement speed of the bit must be kept low, and the pattern may have to be milled in several runs to achieve the required depth. In such cases, the time to machine the microfluidic device necessarily lengthens, sometimes to more than 60 min. However, desktop micromills are relatively cheap, costing ~ £2 k–£10 k, depending on specification.

Laser micromachining of thermoplastics may be performed by lasers covering wavelengths from UV to thermal IR (8–12 µm wavelength). For example, excimer lasers, using step-and-repeat mask illumination, can produce channels with near-vertical sidewalls in thermoplastics (Gower 2001), mainly by photoablation and photodecomposition of the material, from the high UV photon energy. This method produces very little heat and thermal stress in the substrate, and very little re-deposition of ablated material. However, the micromachining process is slow, with only a few µm depth removed per laser pulse. Therefore, a complex pattern may need several hours process time, and, furthermore, the laser equipment will cost > £100 k.

Conversely, 10.6 µm wavelength CO_2_ lasers can pattern sub-mm width microchannels in thermoplastics and silicones at high speed, allowing for rapid change between CAD design patterns, and at relatively low equipment costs of ~ £8 k–15 k for desktop style equipment. This makes CO_2_ laser micromachining advantageous for rapid prototyping of microfluidic devices in the laboratory. Material removal is by thermal ablation, which does have the drawbacks of inducing a heat affected zone (HAZ layer) around the channel, with raised channel edges, and re-deposition of material on the substrate. The latter two factors do make bonding of lids or bases onto the channels more problematic, but this can be alleviated by using solvent vapour assisted thermal bonding. Here, the lid or base, and/or the microchannel patterned substrate are exposed to a solvent vapour e.g. CHCl_3_ (Mohammed et al. 2015; Sun et al. 2015), to soften them, and then pressed together, preferably, in a temperature and pressure controlled hot embosser, which strengthens the bond, while driving off excess solvent.

The aim of the current work is to more fully describe how sealed microfluidic channels may be fabricated in PMMA. This includes demonstrating fabrication of snake channel restrictors to control hydraulic resistance in microfluidic channels. Different flow restrictions between 2 fluid inputs e.g. a seawater sample, and an acid for dissolved CO_2_ liberation, will enable control over metering ratio of the 2 liquids, and improved analyte precision. This may be achieved by various combinations of asymmetric Y meters and snake channel flow restrictors (Tweedie et al. 2020b). Performance of various lengths of flow restrictors will be demonstrated graphically.

## Materials and methods

Cast PMMA (acrylic or Perspex) pre-cut sheets of 100 mm × 100 mm, and various thicknesses, were sourced from Cut Plastics Ltd. The protective cover layer was peeled off the surface before laser engraving. Chemicals (IPA for cleaning, CHCl_3_ for vapour treatment) were sourced from Sigma Aldrich UK Ltd.

A VLS 2.30 Desktop Series, 25 W, 10.6 μm wavelength CO_2_ laser system, manufactured by Universal Laser Systems, was used for engraving Y junctions and various channels in PMMA. The maximum scan speed of the laser head is ~ 1.2 m s^−1^. Available lenses were a 1.5″ standard focal length, and a High Power Density Focussing Optics (HPDFO) lens for highest resolution laser engraving. Maximum resolution was 1000 pulses per inch (ppi). AutoCAD (Educational license) was used for preparing drawings for engraving, via the supplied ULS Control Panel software. Microfluidic channels may be patterned by raster or vector scan, where vector scans are faster, because the laser scans directly only along the lines to be patterned. The laser beam can be defocussed in 0.1 mm increments over a wide range to pattern wider shallower channels, which may be advantageous in certain circumstances (Hong et al. 2010; Prakash et al. 2016; Prakash and Kumar 2017; Matellan and del Río Hernández 2018).

A Roland Modella micromill, MDX-20, was used to mill ¼–28 UN fluidic connections to the rear of 10 mm PMMA substrates, before laser micromachining channels on the front. The screw threads were hand tapped before bonding lids to substrates. Substrates were subsequently cleaned using ultrasonic cleaning in a solution of DI water and IPA, rinsed in DI water, and dried in nitrogen.

Optional smoothing of laser micromachined channels was carried out by exposing them to CHCl_3_ vapour at a height of 4 mm, in a closed petri dish, for 10–12 min. The absorbed vapour was driven off by heating in an oven at 60 °C, for several hours.

Initial flow testing used channels sealed to flat bases via double sided tape (3 M 9087, white with PVC carrier and with modified acrylic adhesive). This is sufficient for straightforward single use tests. Subsequently, vapour assisted thermal bonding of flat 3 mm thick bases to channelled substrates was carried out by exposing the base to CHCl_3_ vapour under the same conditions as for channel smoothing, but for 15 min. The channelled part was then pressed against the softened base, and the 2 parts compressed in a clamp, with the clamp being released, moved, and re-clamped, progressively across the surface, until the whole interface was satisfactorily bonded. This was then placed on a hotplate at 60 °C for ~ 1 h to drive off excess vapour. The channel edge quality of such bonds is sufficient here for flow testing. A loss in channel depth of ~ 25 µm can be expected due to the softened PMMA on the base being pressed down into the channel (Sun et al. 2015).

A DekTak XT stylus profilometer was used for measuring channel profiles, where practical. A Reichert optical microscope with micrometer Z stage was used for measuring channel depths optically, if required. Optical bitmap images of channel plan views and cross-sections were acquired using a 5 Megapixel DinoEye AM7023 digital eyepiece with this microscope. Alternatively, 3 Megapixel images were captured using an Aven digital microscope.

**Fig. 1:**
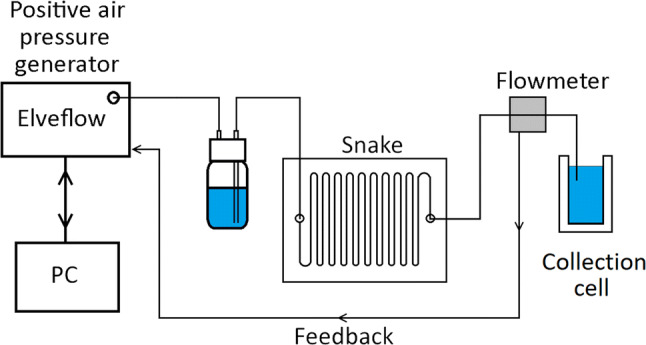
Positive pressure schematic for flow rate testing, with flow sensor feedback

An Elveflow positive pressure generator, AF1-P-1600, was used to pressurise fluid in bottles for flow testing in snake channels, along with Elveflow flowmeters with maximum ranges of 1–5 mL/min, Fig. 1. Flow tests were performed in constant pressure mode, as controlled by the Elveflow Smart Interface software (V2.3.3).

## Results and discussion

### Raster channels, 1.5″ lens

**Fig. 2:**
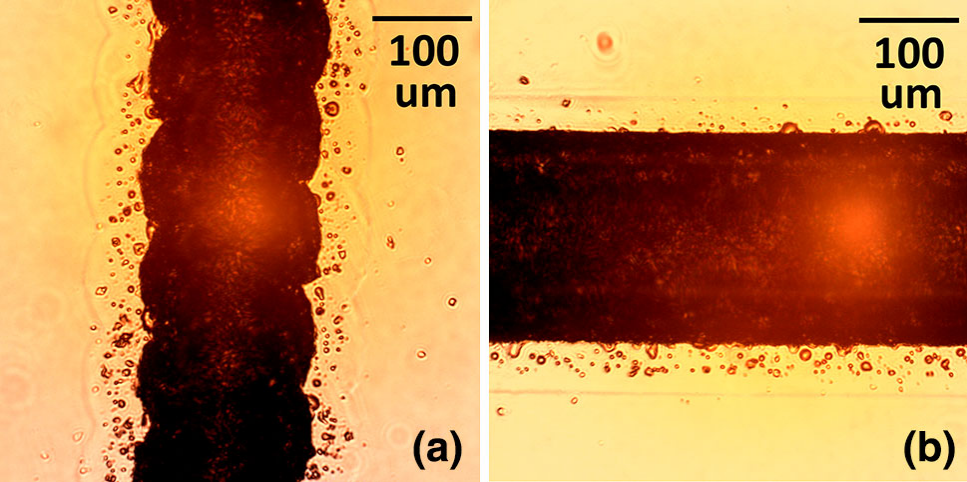
Images for raster written channels in back illumination. The vertical direction channels show edge scalloping not seen on the horizontal direction channels, this being directly produced by the raster scan

The 1.5″ lens was used to create channels via raster, as shown in Fig. 2. This standard lens has a nominal spot size of ~ 75 μm. Vertical lines have scalloped edges, but horizontal lines have much smoother edges, as shown. A minimum power greater than 12.5%, at 100% speed, was required to prevent the scanned line from breaking up into individual points. This can happen due to the interplay between the ablation threshold and the pulse frequency and scan speed of the laser, where there is no direct independent control over these two laser parameters. Re-deposited material is visible as dark edged spots along the channel tops. The amount of re-deposition increases with channel depth. The base is not transparent in back-illumination because of the surface roughness.

**Fig. 3:**
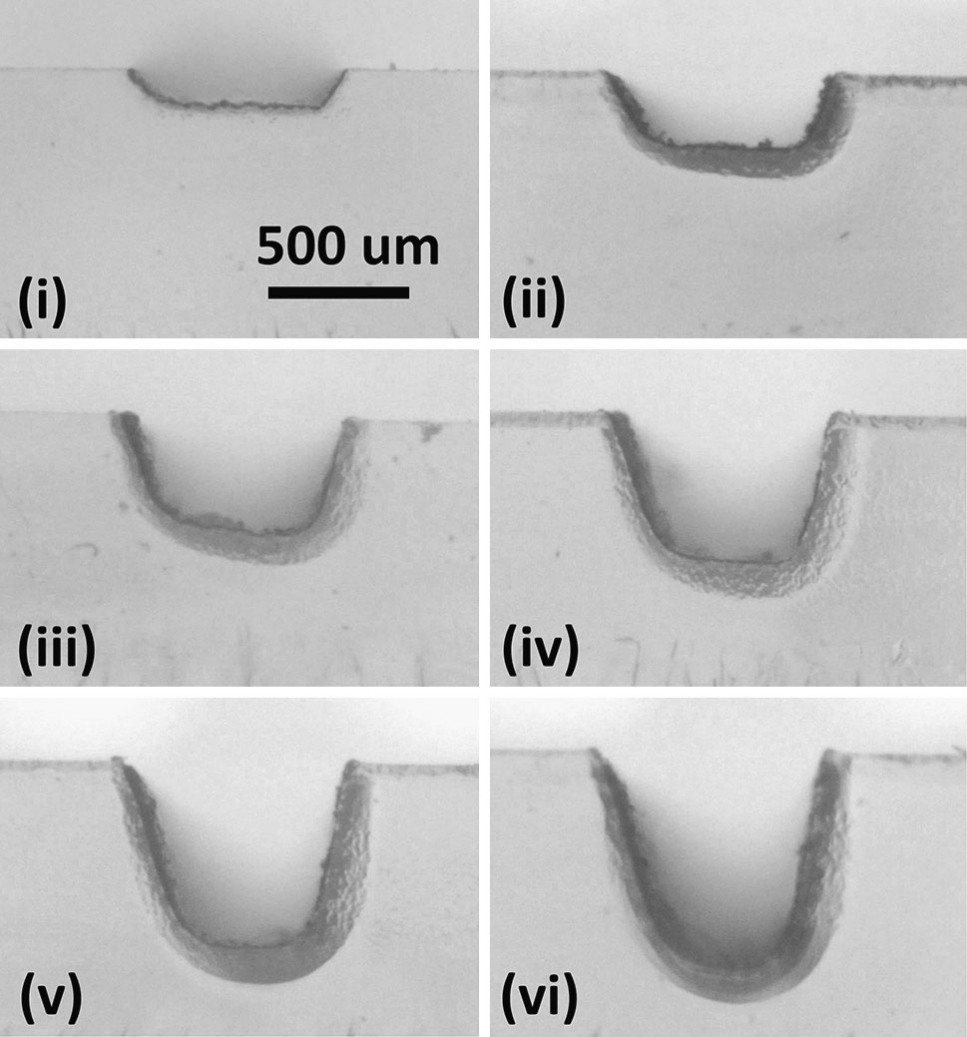
Raster engraved channels via multiple laser passes, at focus, with backlighting. Parts (**i**) to (**vi**) are sequentially engraved for 1X, 2X, 3X, 4X, 5X and 6X times, with the same settings (power 50%, speed 50%). All images are to the same scale. The HAZ is visible as a darkened region surrounding the engraved channels

Test channels were created at laser focus by raster, using an increasing set of multiple runs, from 1 to 6—see Fig. 3. Apart from the shallowest channel of ~ 125 μm, which has a relatively flat base, the rest are U-shaped in cross-section. The images are obtained with backlighting, which shows the well-known Heat-Affected Zone (HAZ) as a darkened region surrounding the engraved features. Another set of channels (not shown) were created with the laser being 3 mm out of focus.

**Fig. 4:**
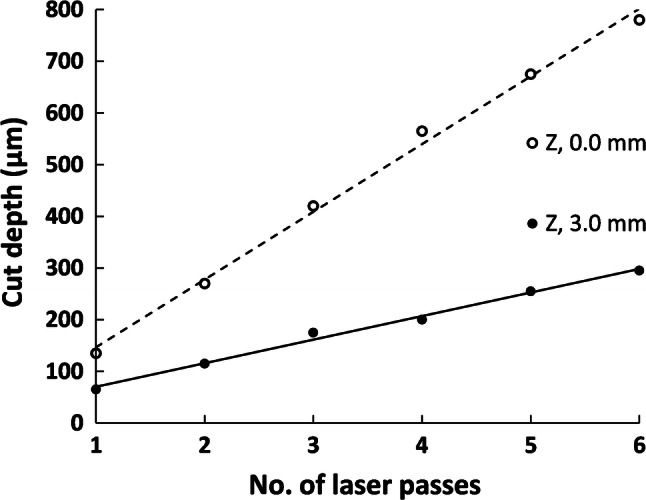
Channel depths for multiple number of laser passes, at 50% power, and 50% speed, for a focussed and 3 mm defocussed beam

The channel depths were measured from the images, for both the focussed and 3 mm defocussed laser, see Fig. 4. A linear increase of depth with number of passes is observed. However, it is clear that the laser focus needs to be correct to within a fraction of 1 mm on the object surface, for repeatable channel depths.

**Fig. 5:**
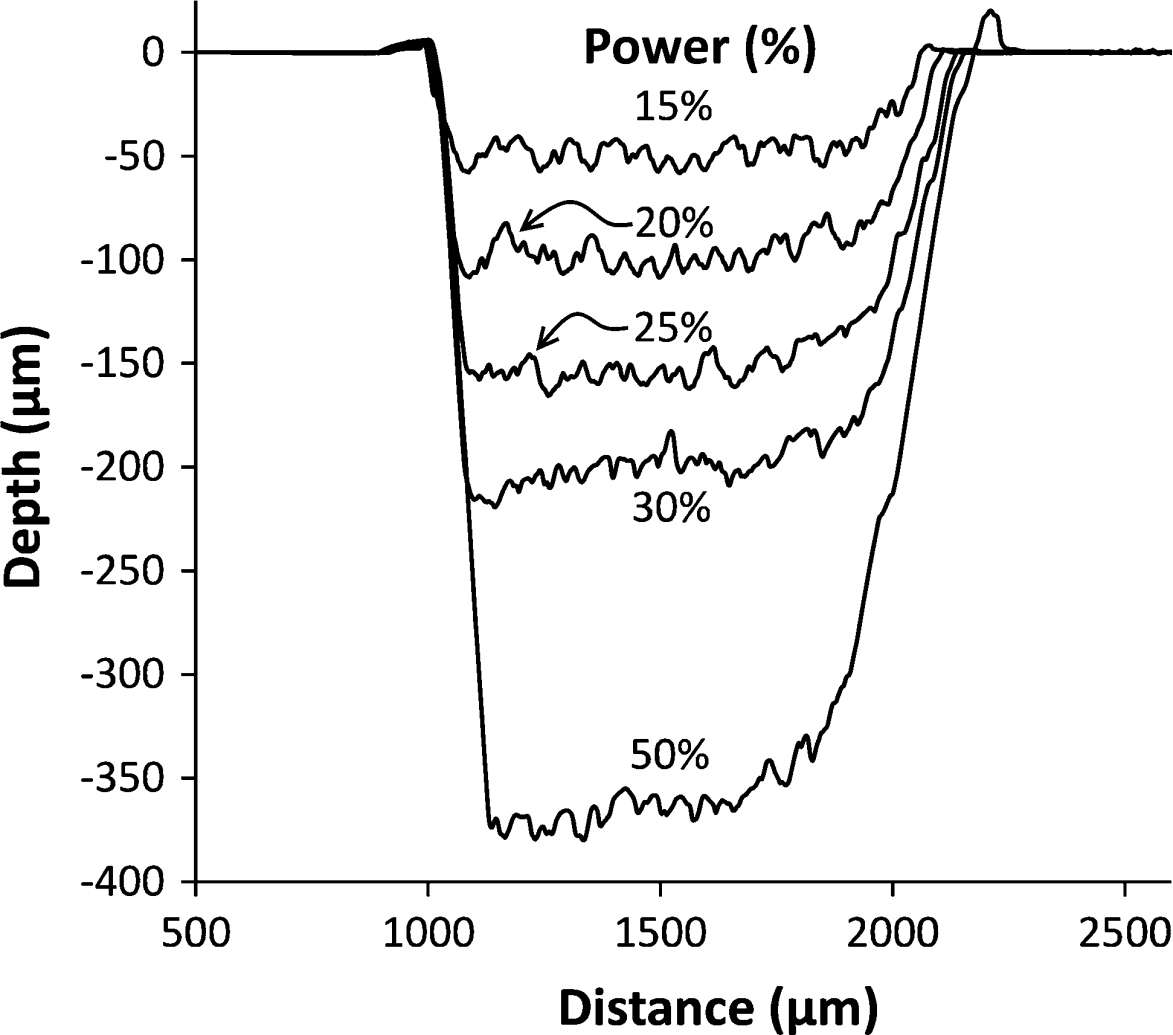
Stylus profilometry scans of raster engraved channels, at 25% speed. Laser Power is indicated as percentage of peak power

A set of ~ 1 mm wide channels were then patterned by raster, at 25% laser speed, and various powers. These were scanned by stylus profilometry, Fig. 5. Slightly raised edges can be seen at the channel tops, and are characteristic of the thermal melt removal process. The apparent channel base profile starts to rise beyond the mid-point, the degree increasing with depth, similar to the asymmetry in Fig. 3, except reversed due to the scan direction. A minimum useable depth of ~ 50 μm was observed, partly because of the base roughness.

**Fig. 6:**
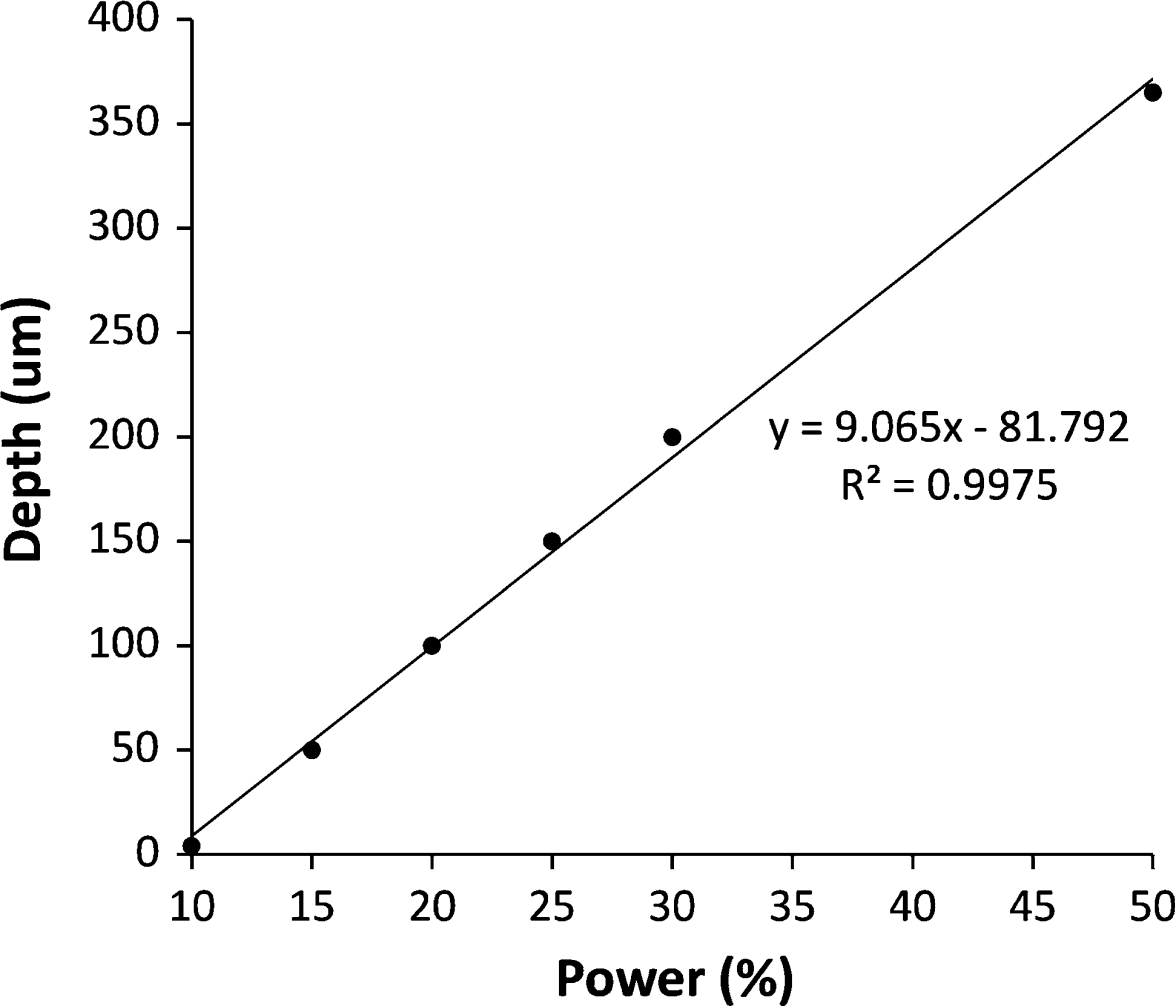
Depth versus percentage laser power, at 25% speed, derived from profilometry scans. A threshold power for engraving, of just under 10% (2.5 W), is evident for this speed and lens

The standard deviation over the flat first half of the channel for 15% power is 5.2 μm, of similar magnitude to previous reports (Mohammed et al. 2015), for single pass CO_2_ laser channel engraving. The channel depths measured for this data show a close to linear relationship with laser power up to 50%, Fig. 6.

**Fig. 7:**
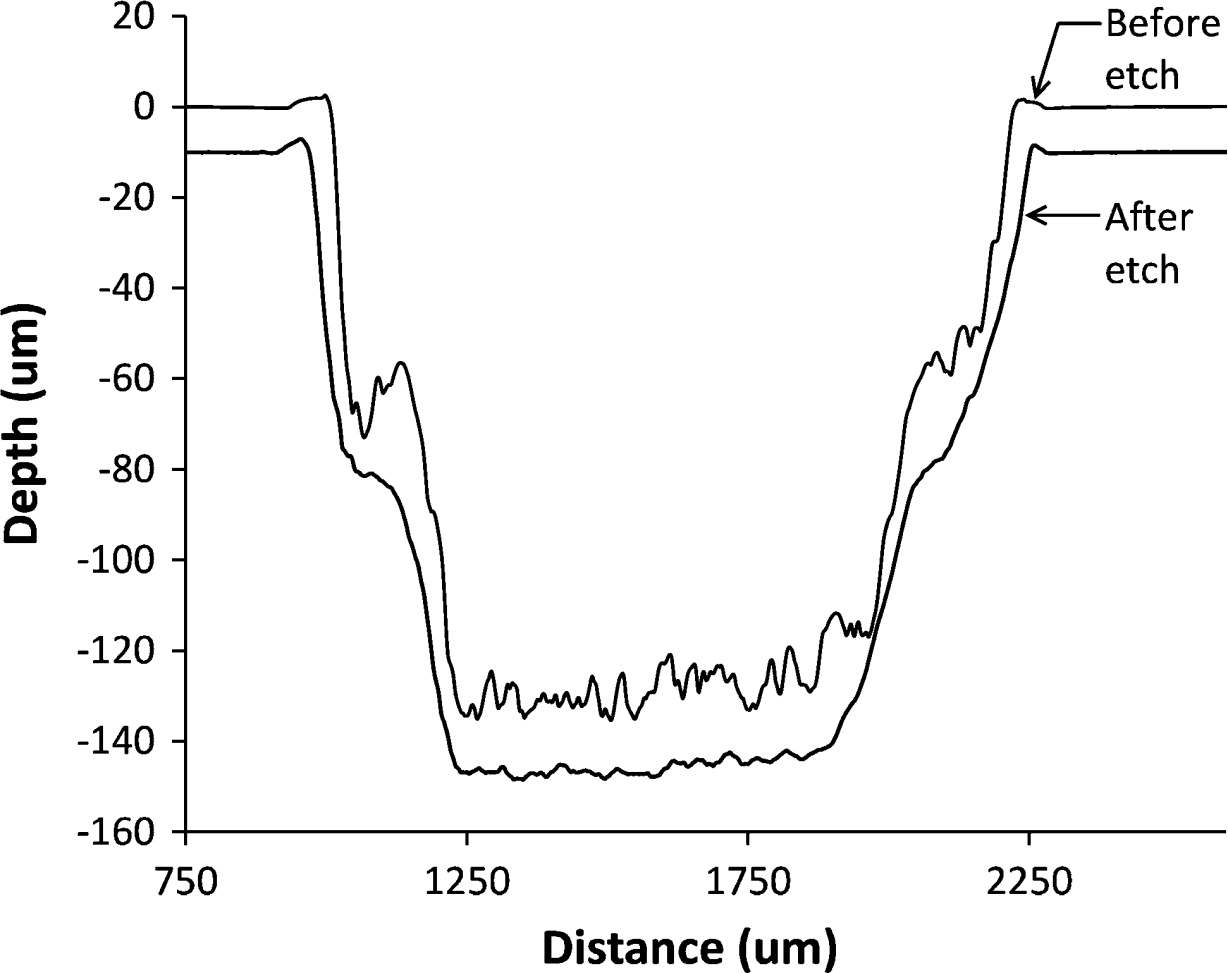
Comparison of ~ 1 mm wide raster engraved channel profile before and after 70% IPA etch for 10 min at 60 °C. The channel was written using 16% power at 25% speed, in 2 exposures

Additionally, some channels raster patterned by 2 runs (each of 16% power and 25% speed) were subjected to a 70% IPA etch for 10 min at 60 °C. This resulted in considerable channel smoothing as shown in Fig. 7.

The standard deviation over the flat first half of the channel base are 2.57 μm before etch, and 0.75 μm after etch. Note that the standard deviation for the 15% power channel, at 25% speed, in 1 run, was 5.2 μm, over one half width, as in Fig. 7. The fact that a double exposure gives a lesser roughness of 2.57 μm is consistent with previous literature work (Mohammed et al. 2017). Also, solvent vapour exposure is known to reduce surface roughness further to ~ 0.7 μm (Mohammed et al. 2017) However, this process needs further optimisation, as a noticeable amount of surface roughening and etching was visible outside the channels, enough to render patches semi-opaque, and possibly to interfere with bonding.

### Vector channels, 1.5″ lens

**Fig. 8:**
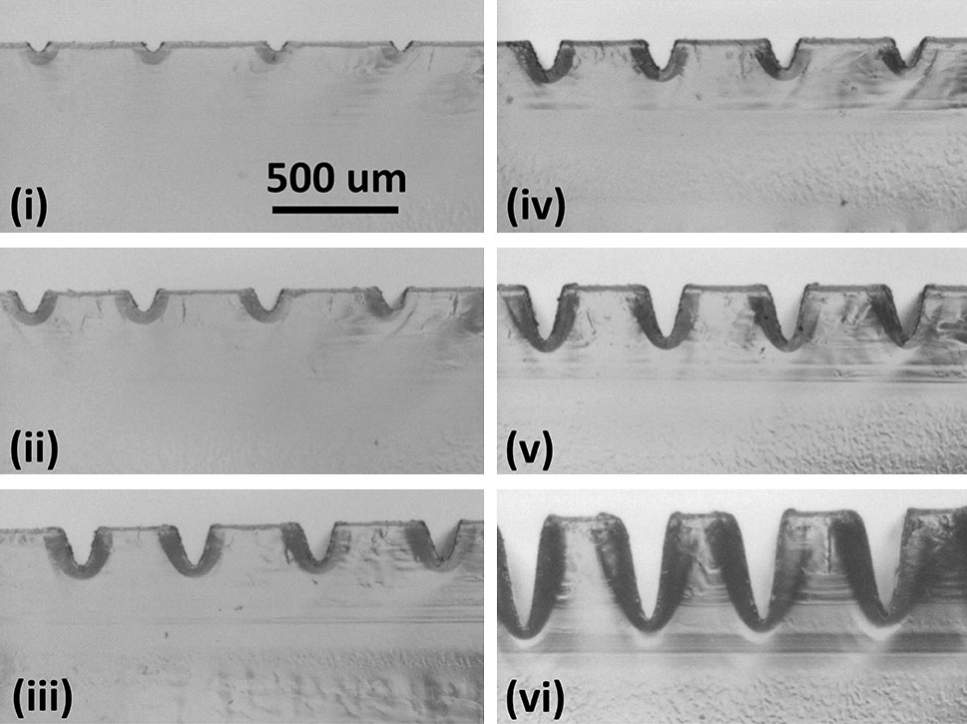
Vector written channel cross-sections, for power of 10%, at speeds of **i** 100%, **ii** 50%, and **iii** 25%, and for powers of 25%, at speeds of **iv** 100%, **v** 50%, and **vi** 25%. All images are at the same scale. The HAZ layer can be seen as a darkened region around the channels

The 1.5″ lens was used to create channels in vector mode, this being achieved by setting a low value for linewidth in Adobe AutoCAD. The laser was observed to give scalloped edges on horizontal X direction channels, but straighter edges on vertical Y direction channels, the opposite to the case for raster scanning. For best results, the channels were written in the Y direction. Note that the channel width changes more noticeably with depth for vector-written channels than for the wider raster-scanned channels. Also, vector-written channels more closely approximate V-grooves, Fig. 8, than U-shaped raster-written channels.

**Fig. 9:**
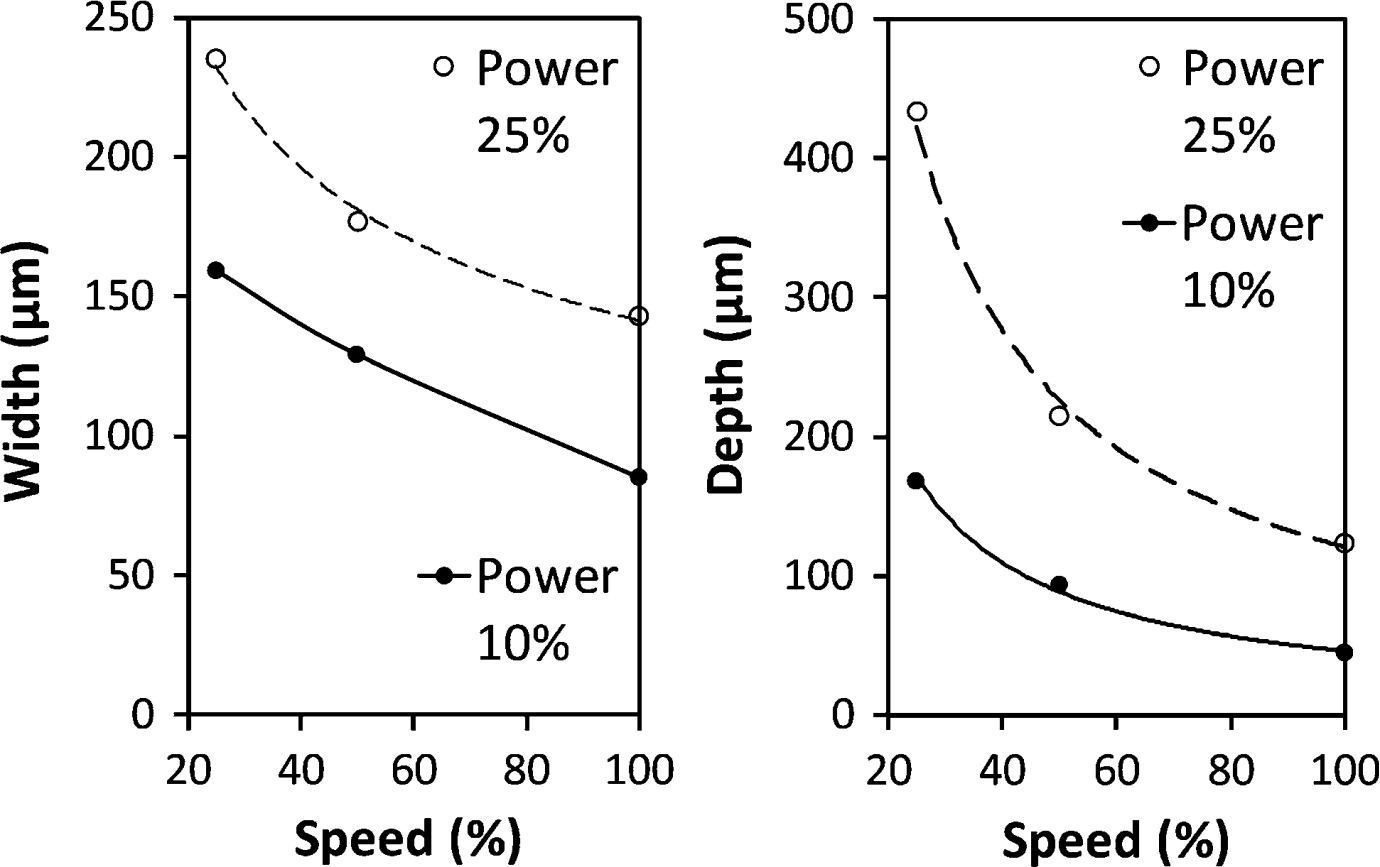
Vector channel width and depth versus speed, for powers of 10% (2.5 W) and 25% (6.25 W)

The channel widths and depths were measured from the photomicrographic images in Fig. 8—see Fig. 9. Note that, because of the V-groove profiles, the stylus profilometer was not used for this case, as it cannot reach to the channel bases, except for shallow profiles.

A minimum width of ~ 80 μm is achieved, but with a depth of only 50 μm. Attempts at achieving shallower depths were found to give line break-up, with large variations in channel depth and width, as the engraving was dominated by the individual laser pulses, which no longer overlapped enough for continuous lines.

**Fig. 10:**
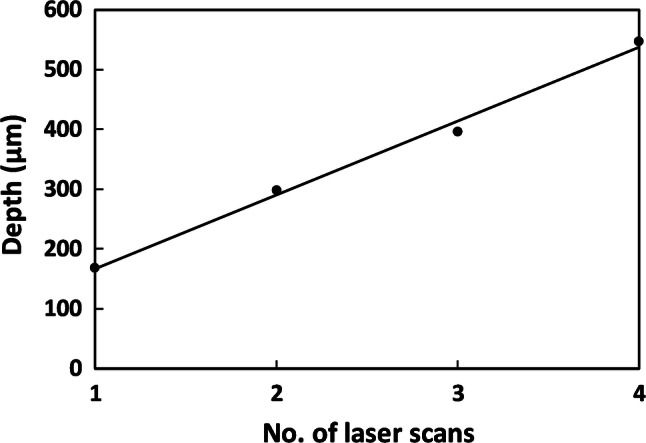
Vector-written channel depths at power 10%, speed 25%, versus number of laser scans for 1.5” lens

Multiple laser passes were also performed at 10% power and 25% speed. As for raster engraved channels, the cut depth increases linearly with number of laser scans, as in Fig. 10.

### HPDFO lens, raster and vector engraving

**Fig. 11:**
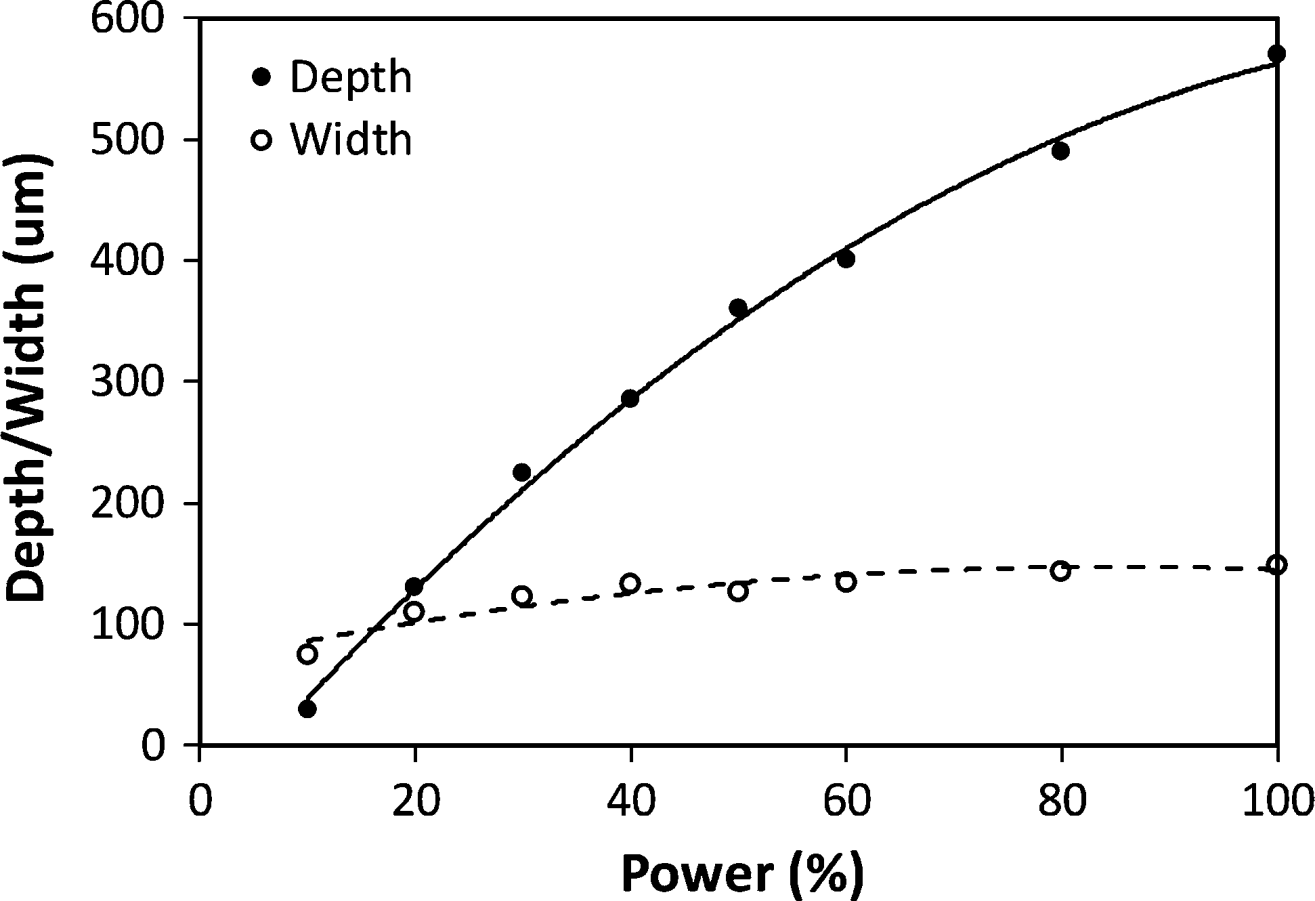
Raster written channels, depth and width variation with laser power at 25% speed, for HPDFO lens

**Fig. 12:**
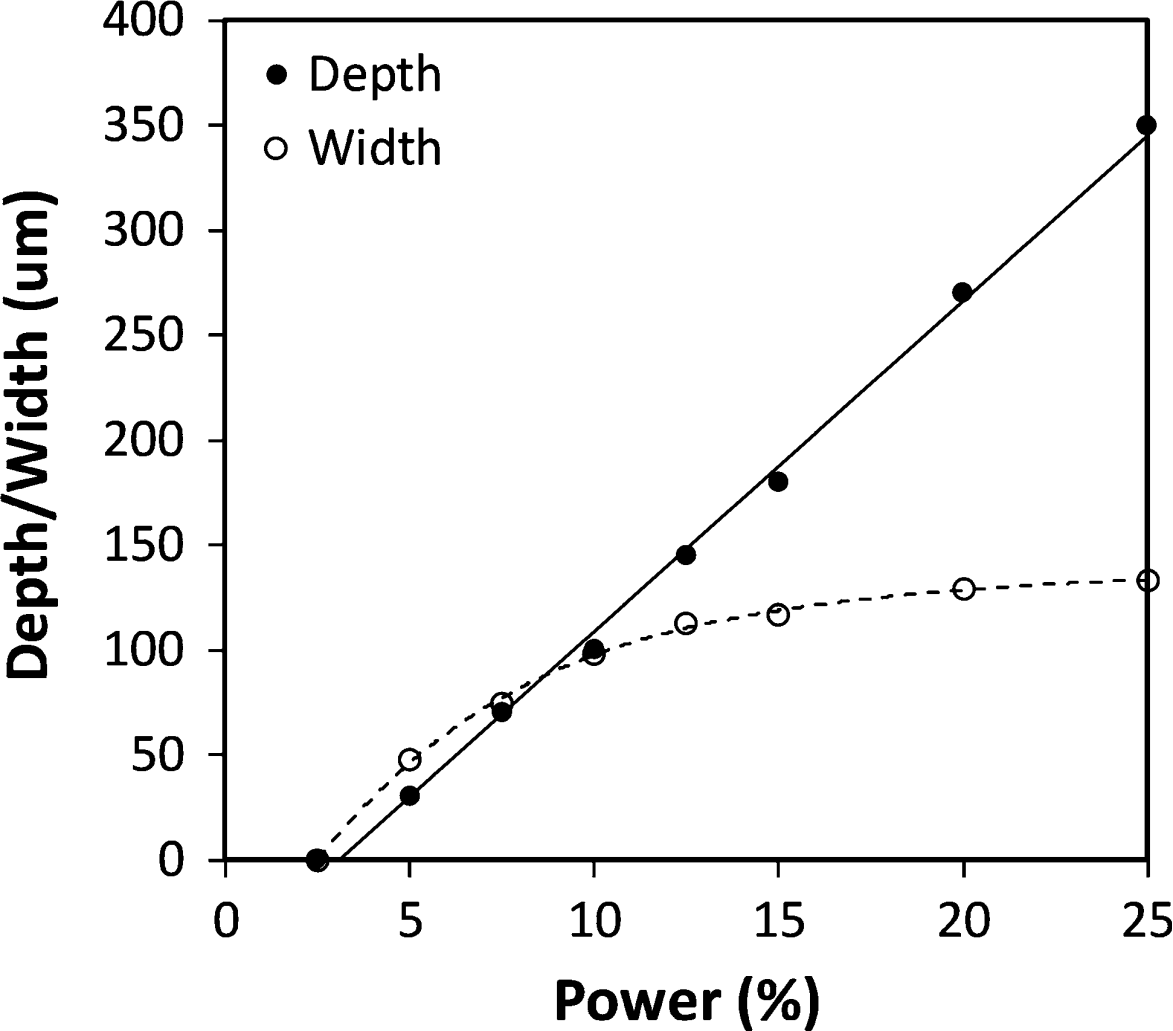
Vector written channels, depth and width versus laser power, at 25% speed, before bonding, for HPDFO lens

The HPDFO lens has a nominal spot diameter of ~ 25 μm, compare to ~ 75 μm spot diameter for the 1.5″ lens. However, for raster-written channels, Fig. 11, the HPDFO lens gave a minimum spot width of ~ 80 μm, similar to the 1.5″ lens, with a depth of ~ 30 μm,

For vector-engraved channels, a width of ~ 50 μm was measured, with a depth of ~ 30 μm, Fig. 12, but with significant width and depth variations, suggesting engraving was not reliable. Therefore, a spot size close to 25 μm may only be achievable for materials with a much lower ablation threshold.

### Defocus effects with HPDFO lens

**Fig. 13:**
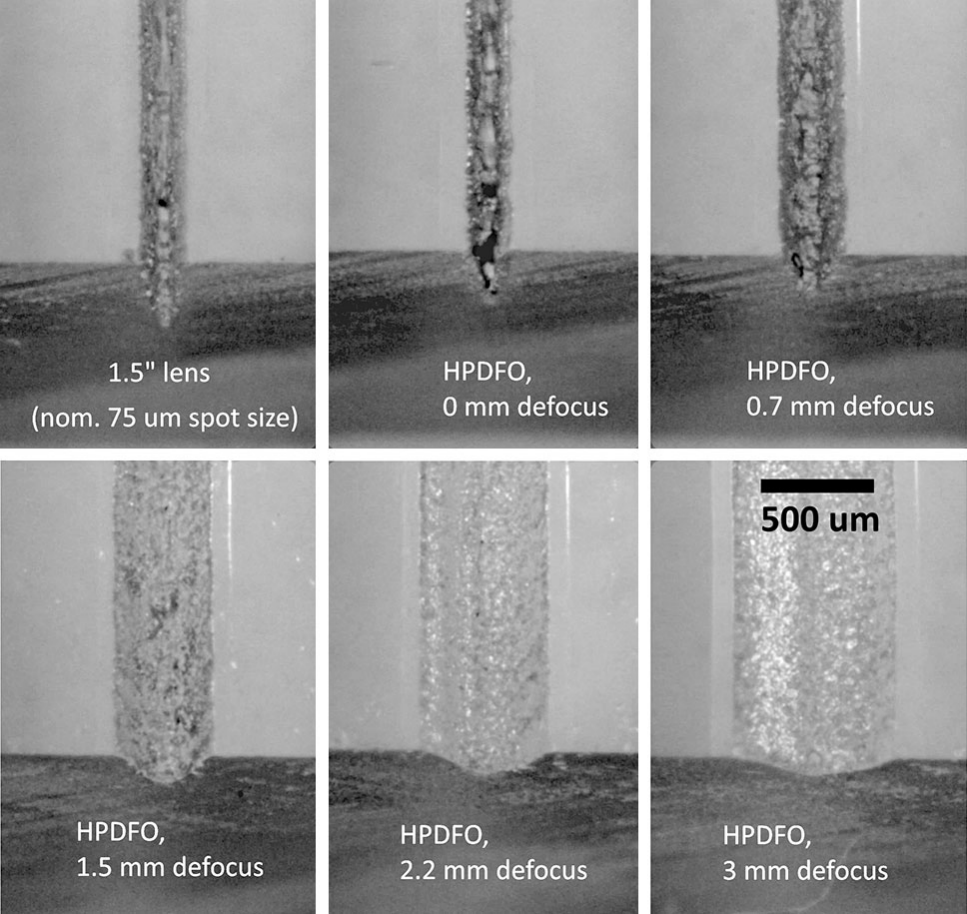
Images of HPDFO lens vector written channels at various defocus settings, with a VLS4.60 50 W CO_2_ laser (samples courtesy of Universal Laser Systems GmbH, Europe)

Defocusing the laser is a method sometimes used to write wider shallower channels in vector mode, as this is much quicker than raster mode. A sample of PMMA with vector engraved channels written by a HPDFO lens was provided on request by the laser manufacturer, Universal Laser Systems (Europe), using a model VLS4.60 50 W CO_2_ laser of 10.6 µm wavelength, at 15% power (7.5 W), and 20% speed, at 750 ppi, see Fig. 13.

**Fig. 14:**
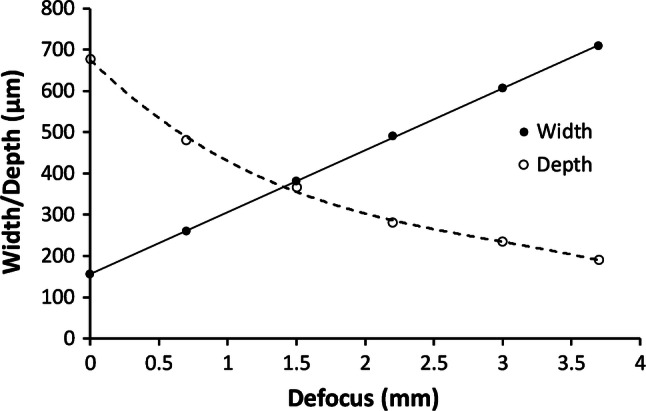
Variation of depth and width with defocus for HPDFO lens (50 W max. power, at 15% setting, 20% speed, and 750 ppi.)

A DekTak XT stylus profilometer could only measure the width and depth of the 3 most shallow channels. For narrower deeper channels, the stylus can only measure the width, but cannot reach the base. However, the depth was then measured from optical images of cross-sections, or via a vertically calibrated travelling microscope, see Fig. 14, which shows a graph of the channel width and depth versus defocus amount for the HPDFO lens. The line width is seen to increase in a highly linear manner with defocus, whereas the depth drops off more rapidly initially.

The line width linear changes with defocus are expected, using the first order geometrical ray tracing approximation, when the optical system is not limited by aberrations or diffraction. The example shows channel depths of up to almost 700 μm at zero defocus with a width of ~ 165 μm. The depth falls to ~ 180 μm at 3.7 mm defocus, with a ~ 680 μm width. It is important to keep channel depths consistent in most microfluidics systems to limit carryover, where fluid from a shallow channel can flow, after a junction, over the top of fluid in a deeper channel, because of laminar flow conditions.

### Bonding

**Fig. 15:**
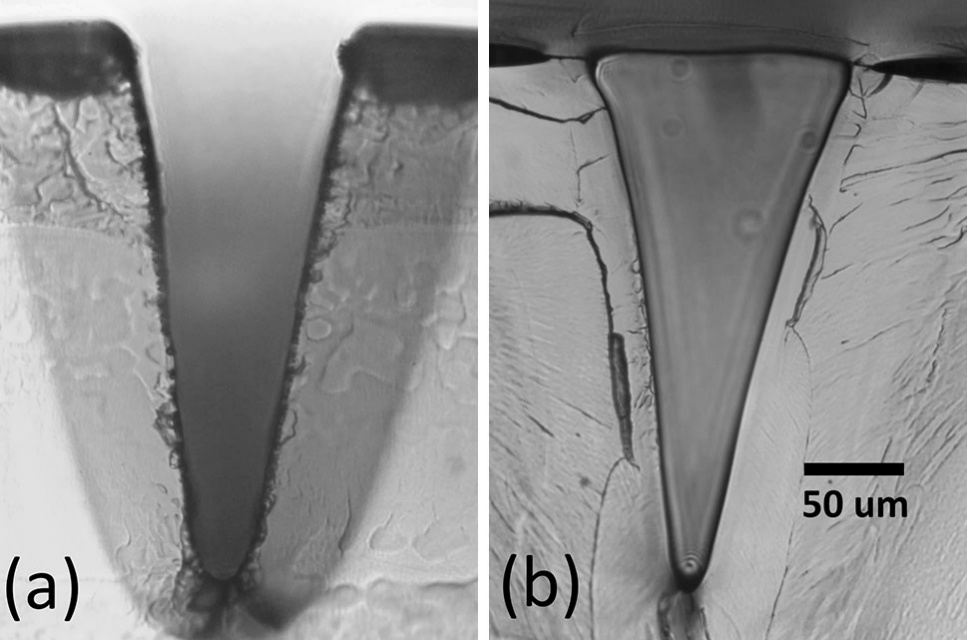
Cross-sections of V-groove vector engraved channels, using 25% power (6.25 W), and 25% speed (~ 0.3 m s^−1^) **a** before bonding, and **b** after bonding

Chloroform vapour assisted bonding was performed for V-groove vector-written channels, and photomicrographs imaged for characterisation. An example of a V-groove written at 25% power (6.25 W) and 25% speed (~ 0.3 m s^−1^) is shown in Fig. 15. Generally, around 25 µm depth is lost in the bonding process, due to reflow of the softened PMMA into the channel (Sun et al. 2015).

**Fig. 16:**
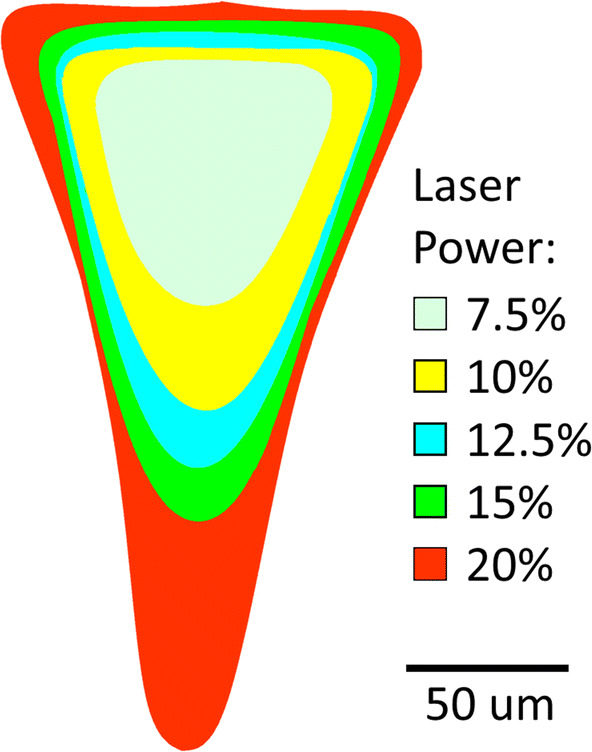
Traced contours (from images) of vector engraved channel cross-sections, versus laser power (%), where the peak power was 25 W, and laser speed was 25% (~ 0.3 m s^−1^)

Closed contours were fitted onto digital photomicrographs of bonded microchannels, which had been fabricated for different powers at 25% laser speed, Fig. 16. The channel shape, while being generally described as V-grooved, does become increasingly steep-walled as the laser power increases. Despite the gradual change in profile, the cross-sectional areas increase approximately linearly with laser power up to 25%, as previously reported (Tweedie et al. 2020b).

### Snake channel devices

**Fig. 17:**
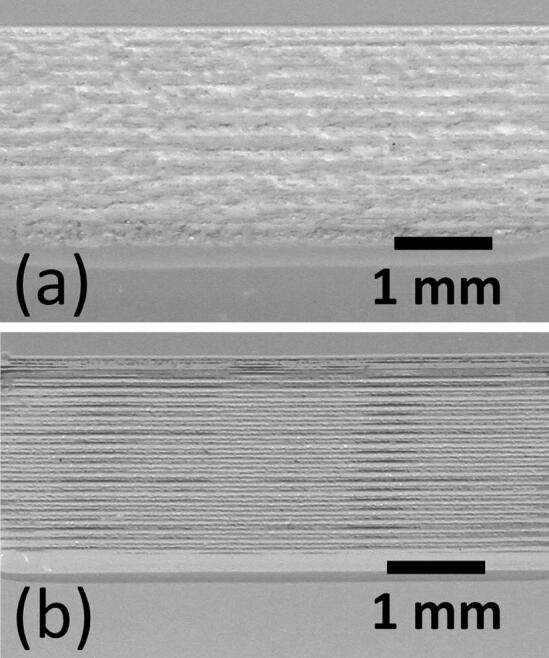
**a** Channel in PMMA written by raster scan, **b** raster engraved channel after smoothing by CHCl_3_ vapour treatment. The channel has improved transparency afterwards, but the laser scan lines are more evident

**Fig. 18:**
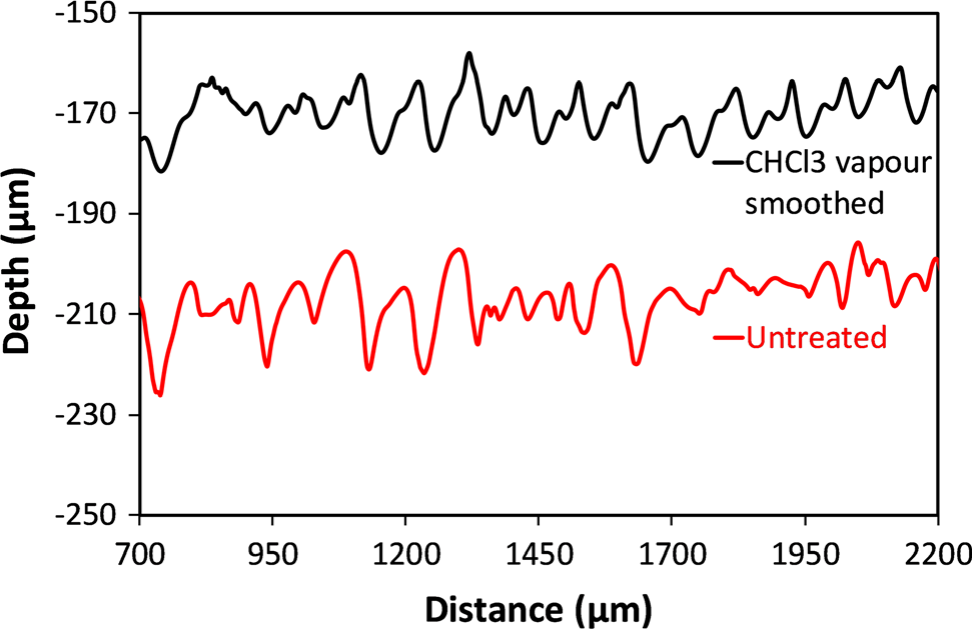
Stylus profilometry scans perpendicular to the long axis of the channels. Chloroform vapour smoothing slightly sharpens larger scale features related to raster line writing, with little change in the large scale surface roughness. Data sets offset for clarity

Initially, wide shallow channels were fabricated in raster mode. We tested channel smoothing by CHCl_3_ vapour treatment (Ogilvie et al. 2010), and found that this increased the optical clarity significantly, Fig. 17, although this is because the vapour smooths out roughness on the scale of light wavelengths (400–700 nm). However, it does not smooth out features to the same degree on larger scales, as can be seen from the remaining laser scan lines in Fig. 17, and the stylus profilometry scans in Fig. 18.

The rms roughness of the channels are 5.27 μm before CHCl_3_ vapour treatment, and 4.17 μm afterwards. In fact, the definition of the laser scan lines is slightly enhanced by the CHCl_3_ treatment, as evidenced by the sharper peaks in Fig. 18, and by the clearer optical definition in Fig. 17. This is noticeably different to the case of heated 70% IPA smoothing, where a significant amount of material is removed.

**Fig. 19:**
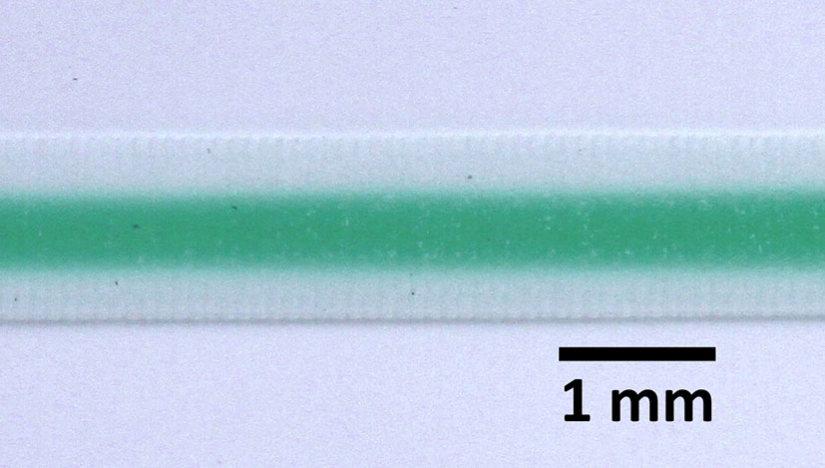
Raster engraved snake channel showing fresh dye input flowing through the centre of the DI prefill. At least ×2 the snake volume is required to clear away the previous fluid

A device with 2 inputs meeting at a Y junction, which then led into a snake channel region, was fabricated with raster engraved channels. This was bonded via double-sided adhesive tape to a PMMA base, and tested for microfluidic flow using DI water and aqueous diluted filtered green food dye. It was observed that when flowing dye into the channel, which was prefilled with DI water, the dye did not uniformly displace the water, but actually flowed initially through the centre of it. This is shown clearly in a magnified view in Fig. 19. It was found that at least 2 to 3 times the channel volume needed to be introduced to fully clear out the previous water sample. If lesser volumes are input, then mixing between samples occurs, due to carryover of previous samples.

**Fig. 20:**
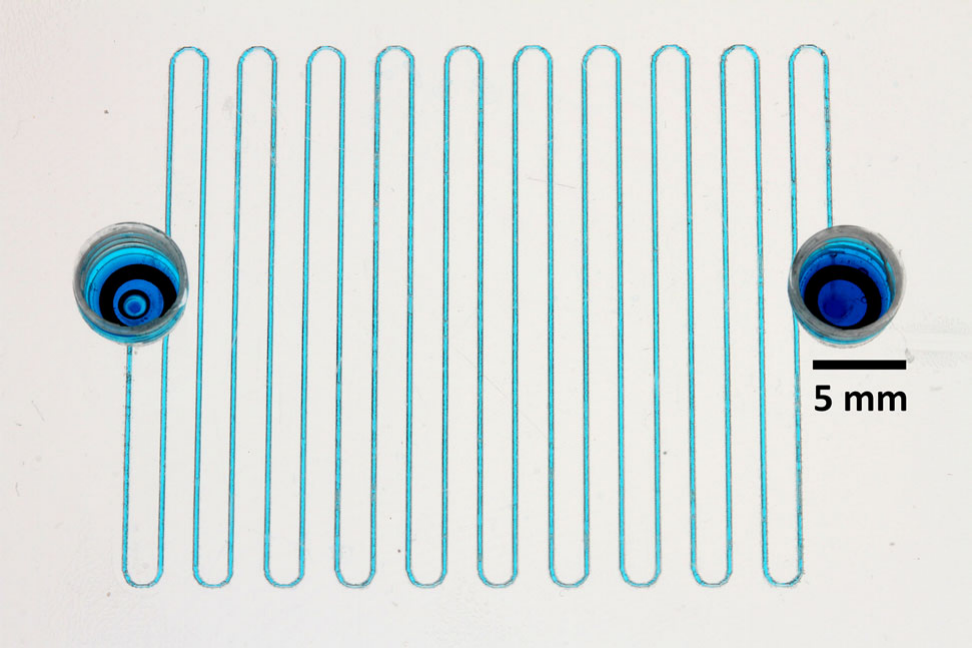
Raster written snake channel with ¼–28 ports, of 663 mm length, fabricated at settings of 100% power, 25% speed, and AutoCAD linewidth of 0.09 mm

Subsequently, a set of snake channels were fabricated in raster mode, at 100% power (25 W) and 25% speed (~ 0.3 m s^−1^). Lengths were 330 mm, 663 mm, 994 mm, and 1320 mm, with the 663 mm device being shown in Fig. 20. These were tested for flow rate versus applied positive pressure, using the Elveflow pressure generator, flowmeters, and control software.

**Fig. 21:**
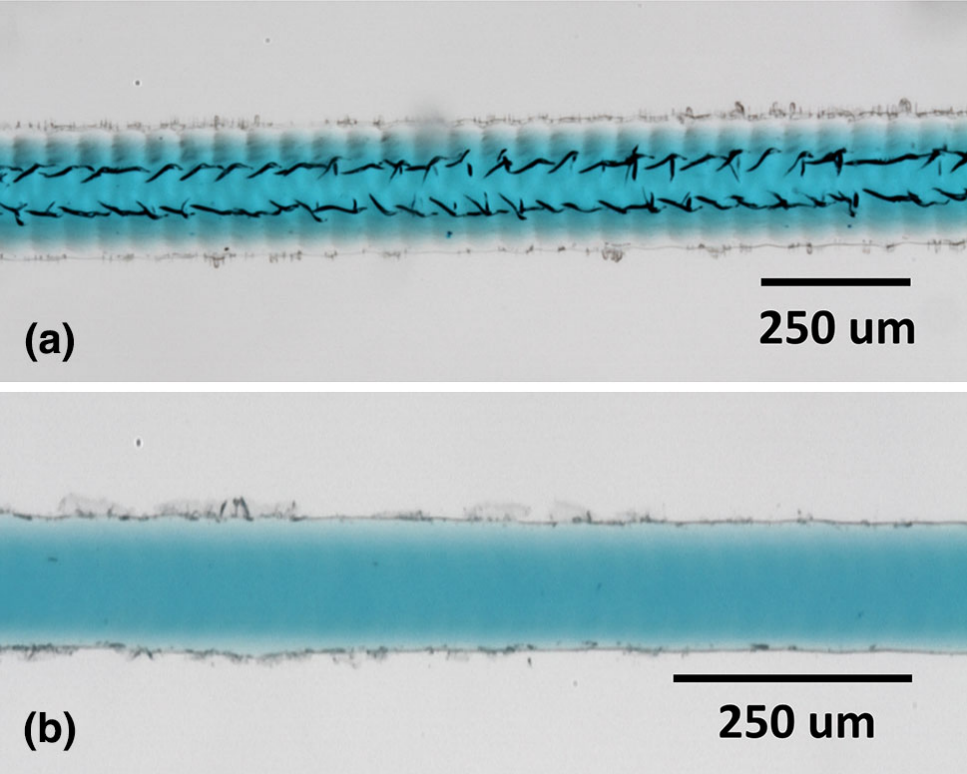
Dye filled channels after CHCl_3_ vapour assisted bonding. In **a** microcracks are clearly visible in the channel when treated with CHCl_3_ vapour before bonding. In **b** microcracks are not visible when only the flat base was CHCl_3_ vapour-treated before bonding

It was found that when snake channels were directly exposed to CHCl_3_ vapour smoothing before bonding, there was a tendency for microcracks to form, particularly, in the base of the channel. These are visible as dark striations in Fig. 21a, and were not removed by driving off excess vapour at 60 °C. In comparison, when the CHCl_3_ vapour is applied to the flat base for bonding, the engraved channel remains free of microcracks, as in Fig. 21b.

The microcracks do not actually impede flow, although some fluid may leak into them, or they may trap very small amounts of air, but these are not serious effects. However, they are visually distracting, and can affect the overall transparency of the channels. To smooth the channels with CHCl_3_ vapour before bonding, but prevent microcrack formation, the PMMA normally needs annealing near to the glass transition temperature (~ 104 °C) of PMMA, either before laser writing, or after CHCl_3_ vapour exposure.

### Y meter fabrication

**Fig. 22:**
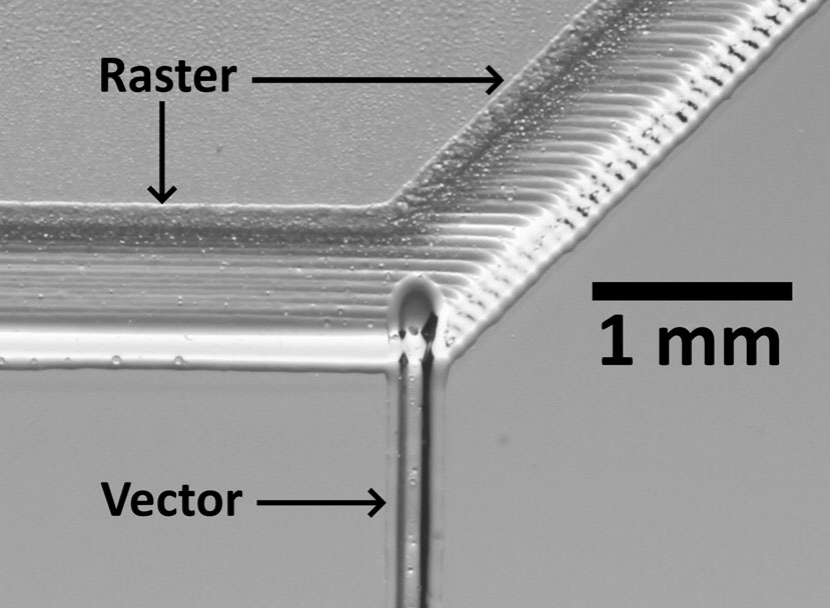
Optical image of a thin vertically oriented vector-written channel, meeting a broader raster-written channel, to form an asymmetric Y junction. All channels engraved by HPDFO lens

Asymmetric Y meter channels, intended for mixing 2 fluids in ratios other than 1:1, were fabricated with wide input and output channels written by raster, as shown in the backlit photograph in Fig. 22. The narrow input was written by vector in the vertical direction, as this avoids issues with scalloping on vector engraved line edges in the X direction.

**Fig. 23:**
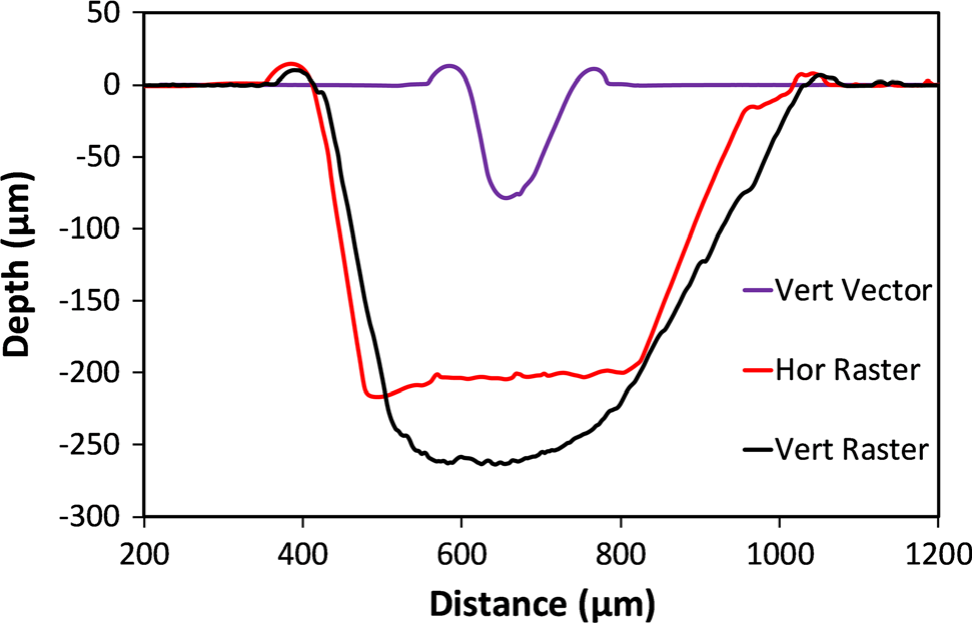
DekTak stylus profiles of typical vector and raster engraved channels using the HPDFO lens

The dark vertical lines on either side of the vector written input channel are an optical effect. The channel lies within the outer edges of the dark bordering lines, and is ~ 135 µm wide, according to the stylus profilometer scan in Fig. 23. This graph also shows some differences in raster engraved channel depth and base flatness between channels engraved in the Y (vertical) direction, and X (horizontal) direction. Here, the horizontal direction channel has a flatter base, while the vertical direction channel is deeper by ~ 50 µm, and has a slight asymmetry, with a shallower gradient on the right hand side.

Although not further tested as complete devices here, a similar asymmetric Y meter, with a base sealed on by vapour-assisted thermal bonding, was used recently for metering HCl acid into 2 mM sodium bicarbonate solution, for laboratory tests on 2 mM CO_2_ measurement by integrated Au electrodes (Tweedie et al. 2020a). This relates to laboratory investigations of dissolved CO_2_ measurement. Comparisons have also been conducted between use of asymmetric Y meters and snake channel for the same application (Tweedie et al. 2020b). This work provides more detail on fabrication issues related to these 2 device types, however, as in Fig. 23.

### Flow testing results for snake channel restrictors

**Fig. 24:**
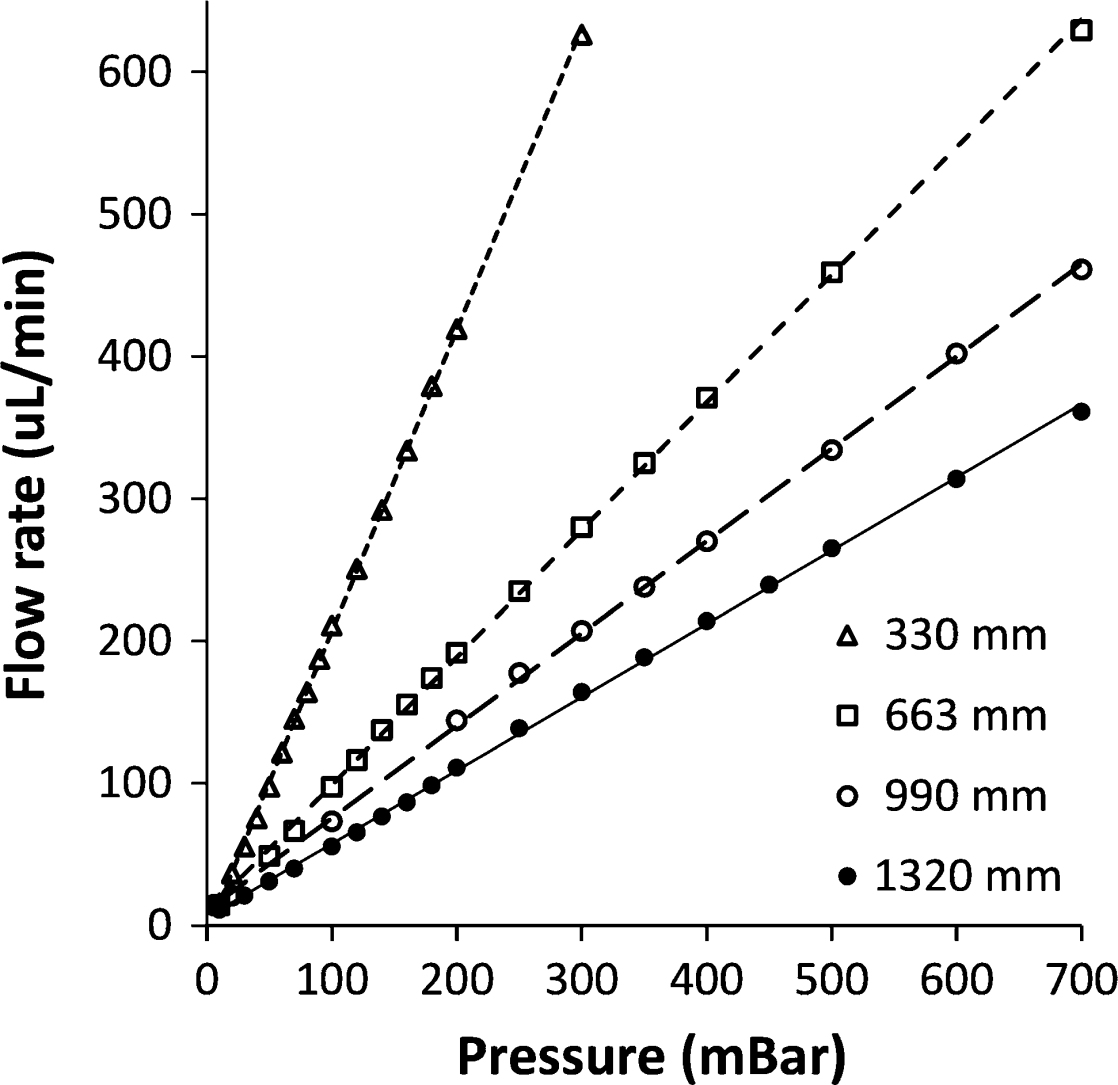
Post-bond flow rates versus applied positive pressure, for 4 different snake channel lengths

The graph in Fig. 24 shows the flow rate versus pressure data for all 4 snake channel devices, tested immediately after initial bonding.

A final overnight oven soak at 60 °C had not yet been performed on these, at the time of testing, to preserve the bond quality at its best condition along the channel edges. It was later suggested that to preserve the quality of the flow characteristics after an overnight oven soak requires that uniform pressure be maintained over the whole device throughout bake-out.

Each device shows a linear characteristic over the range tested, and the gradient decreasing as the channel length increases. This is as expected, since the channel fluidic resistance should increase linearly with channel length.

**Fig. 25:**
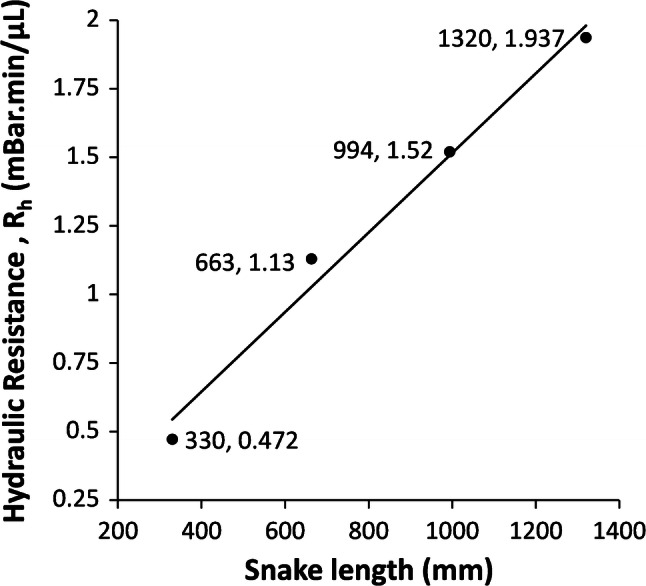
Graph of R_h_ for post-bond snake channels, calculated as the inverse gradients of the flow rate lines

The hydraulic resistance, R_h_, is the inverse gradient of the flow rate versus pressure data. From the data in Fig. 24, the calculated R_h_ versus snake length characteristic is shown in Fig. 25. As expected, R_h_ increases linearly with channel length.

The different R_h_ values achieved for the different lengths of snake channels means that 2 different ones can be used in each of 2 channels to give a meter ratio predicted by the ratio of the R_h_ values, or, indeed, the channel lengths. This can be useful for accurately controlling the meter ratio of 2 channels, when fluid is drawn through both simultaneously using a single pump at the outlet.

## Conclusions

Rapid fabrication of microfluidic channels via a CO_2_ laser using standard 1.5″ and HPDFO lenses was investigated. The nominal laser spot diameters are ~ 75 µm, and ~ 25 µm, respectively. In practise, the minimum continuous channel dimensions in PMMA before bonding, for the HPDFO lens, were ~ 80 µm width, with a 50 µm depth. Defocussing the laser can give shallower broader channels, which has utility in some circumstances.

Raster engraved U-shaped cross-sectional channels have a typical roughness of ~ 5 µm rms. Heated IPA alcohol solution can reduce this to ~ 0.75 µm rms, though the white residue on the top surface reduces optical clarity. CHCl_3_ vapour smoothing increases optical transparency of microchannels, although the large scale roughness only changes slightly from 5.27 μm before CHCl_3_ vapour treatment, and 4.17 μm afterwards. Transparency is not a critical factor in electrically analysing water samples, however.

Sealing of microchannels to bases via CHCl_3_ vapour assisted bonding shows very few solvent-induced microcracks in raster engraved channels, when only the flat base is vapour treated. Micro-cracks are clearly visible if both channels and bases are vapour treated.

The HPDFO lens is useful for defining V-shaped channels in vector mode, although this is best in the Y direction, perpendicular to the raster scan X direction. This avoids scalloping of the vector line and gives a straight edge, but constrains the microfluidic designs. An asymmetric Y meter design was explored, where the narrower channel is defined by a Y direction vector. This is useful for performing metering functions, e.g. when metering a concentrated acid into seawater for CO_2_ release and measurement.

Flow test results were demonstrated for raster-engraved snake channel restrictors, which have applications in balancing meter ratios of fluids in channels. Flow rate in as-bonded channels was proportional to applied pressure, and hydraulic resistance, R_h_, was linearly proportional to snake channel length. This enables layouts to be designed for specific metering ratios of fluids, to cover applications from ocean sensing to biomedical analysis.
